# Nutrient-sensing and mTORC1 regulation in neuronal homeostasis: from metabolic signaling to neurodegeneration

**DOI:** 10.70401/EXO.2026.0009

**Published:** 2026

**Authors:** Sung Min Son, Weining Li, David C. Rubinsztein

**Affiliations:** 1 Cambridge Institute for Medical Research (CIMR), University of Cambridge, Cambridge CB2 0XY, UK; 2 UK Dementia Research Institute, Cambridge Institute for Medical Research (CIMR), University of Cambridge, Cambridge CB2 0XY, UK

**Keywords:** mTORC1, neurodegeneration, nutrient sensing, autophagy, acetyl-coenzyme A, proteostasis, metabolic flux, neuronal homeostasis

## Abstract

Neurons rely on precise nutrient-sensing mechanisms to sustain proteostasis and stress resilience across a lifetime. Among these, mechanistic target of rapamycin complex 1 (mTORC1) functions as a central metabolic hub, integrating amino acid availability, growth factor signals, and energetic status to coordinate protein synthesis, autophagy, and neuronal survival. Neuronal mTORC1 regulation is highly specialised, reflecting unique metabolic demands, axonal compartmentalisation, and dependence on long-term homeostatic control that is not shared by non-neuronal cell types. Beyond canonical PI3K–Akt and AMP-activated protein kinase (AMPK) signaling, emerging evidence highlights metabolic intermediates, most notably leucine-derived acetyl-coenzyme A (AcCoA), as critical upstream regulators that couple nutrient flux to mTORC1 activity via EP300-mediated Raptor acetylation. Chronic dysregulation of these pathways drives persistent mTORC1 hyperactivation, progressive autophagy impairment, and accumulation of proteotoxic species, collectively contributing to neurodegeneration. In Alzheimer’s disease, aberrant mTORC1 activity is linked to tau hyperphosphorylation and amyloid-β accumulation; in Parkinson’s disease, to α-synuclein aggregation and mitophagy failure; in Huntington’s disease, to impaired clearance of mutant huntingtin; and in amyotrophic lateral sclerosis (ALS), to dysregulated proteostasis in motor neurons. This mini review synthesizes current understanding of neuronal mTORC1 regulation, with an emphasis on the AcCoA–acetylation axis as an emerging metabolic control mechanism, its disease-specific implications across major neurodegenerative conditions, and the therapeutic opportunities these insights reveal upstream of mTORC1.

## 1 Introduction

Neurons are uniquely vulnerable to metabolic imbalance. As post-mitotic, highly polarised cells with extraordinary energetic demands, they must maintain proteome integrity and synaptic function over decades^[1,2]^. Unlike dividing cells, neurons cannot dilute damaged proteins through proliferation; instead, they rely heavily on finely tuned proteostatic systems to sustain stress resilience across a lifetime^[3]^. Nutrient- and energy-sensing pathways therefore serve not merely metabolic functions but act as long-term regulators of neuronal survival^[4]^.

Mechanistic target of rapamycin complex 1 (mTORC1) sits at the core of this integration, functioning as a central metabolic hub^[5,6]^. mTORC1 coordinates canonical inputs, including amino acid availability, growth factor signaling, and cellular energy status via AMP-activated protein kinase (AMPK), to regulate protein synthesis, lysosomal biogenesis, and autophagy^[5–8]^. In the brain, mTORC1 responds to neurotrophic factors such as insulin and brain-derived neurotrophic factor (BDNF)^[9,10]^, and, beyond these core metabolic functions, governs processes critical for nervous system development and maintenance, including axonal regeneration and sprouting^[11]^, myelination^[12,13]^, dendritic arborization^[14]^, and synaptic plasticity^[15–17]^. Therefore, neuronal mTORC1 activity must be precisely regulated. Excessive mTORC1 activity promotes anabolic drive, suppresses the autophagic removal of toxic aggregate-prone proteins, and increases proteotoxic stress, impairing neuronal and brain development^[4,18,19]^. Conversely, insufficient activity compromises synaptic maintenance, dendritic spine morphology, structural stability, and neural regeneration capacity^[20,21]^. Thus, neuronal physiology depends not on optimal mTORC1 activity, but on dynamic modulation and precise tuning tailored to developmental stage, neuronal subtype, and subcellular compartment^[17,19]^.

Neurodegenerative diseases, including Alzheimer’s disease (AD), Parkinson’s disease (PD), Huntington’s disease (HD), and amyotrophic lateral sclerosis (ALS), share a striking convergence, including impaired proteostasis accompanied by persistent or maladaptive mTORC1 signaling^[4]^. While their genetic and environmental triggers differ, altered mTORC1 regulation emerges as a common pathogenic feature across multiple neurodegenerative disorders^[4]^. Increasingly, evidence suggests that neuronal mTORC1 regulation is highly specialised, reflecting unique spatial constraints and metabolic demands^[17,22,23]^. Beyond canonical signaling, neurons interpret intracellular metabolic flux, with metabolic intermediates such as acetyl-coenzyme A (AcCoA) acting as critical signaling molecules that couple nutrient flux to mTORC1 activity through post-translational mechanisms, including acetylation-dependent regulation^[17,24–26]^.

Herein, we review our current understanding of neuronal mTORC1 regulation, with a particular emphasis on the metabolic control mechanisms involving the p300–mTORC1–autophagy axis. We explore how these specialised pathways contribute to neurodegeneration and highlight new opportunities for therapeutic intervention aimed at restoring physiological nutrient sensing upstream of mTORC1.

## 2 mTORC1: Pathways and Regulation

### 2.1 The composition of mTORC1

The mammalian (or mechanistic) target of rapamycin (mTOR) is an evolutionary conserved serine/threonine kinase belonging to the phosphoinositide 3-kinase (PIKK)-related kinase superfamily^[5,6]^. In mammals, mTOR is the catalytic core of mTORC1, which comprises two positive and two negative components: the regulatory-associated protein of mTOR (Raptor)^[27,28]^, the mammalian lethal with Sec13 protein 8 (mLST8)^[29]^, the proline-rich AKT substrate of 40 kDa (PRAS40)^[30–32]^, and the DEP domain-containing mTOR-interacting protein (DEPTOR)^[33]^. Among these subunits, Raptor is the principal defining subunit of mTORC1^[27]^ (not present in mTORC2), as it functions as a scaffold that confers substrate selectivity and coordinates the nutrient-dependent mTORC1 translocation to the lysosomal membrane^[6,34–36]^. mLST8 is also a positive regulatory subunit that associates with the kinase domain of mTOR and stabilizes the complex^[6,29,37]^, whereas PRAS40 and DEPTOR act as inhibitors in the complex, with their roles regulated by upstream signals^[6,30–33,38]^.

### 2.2 The upstream and downstream signaling of mTORC1

As the master controller of cellular growth, mTORC1 governs the balance between anabolic and catabolic pathways in response to environmental inputs^[5,6]^. It senses changes in intracellular and extracellular nutrient availability, primarily amino acids^[36]^, together with growth factor signaling^[39,40]^, cellular energy status^[41,42]^, oxygen^[43,44]^, cholesterol^[45,46]^ and glucose^[5,6,47,48]^. Under nutrient-replete conditions, mTORC1 is activated and promotes anabolic pathways by phosphorylating key downstream effectors, including ribosomal protein S6 kinase (S6K), and eukaryotic initiation factor 4E-binding protein (4E-BP), thereby facilitating protein, lipid, and nucleotide synthesis^[6,49]^. Meanwhile, it suppresses catabolic processes such as autophagy and lysosomal biogenesis through phosphorylation of unc-51-like autophagy activating kinase 1 (ULK1)^[50–54]^ and the transcription factor EB (TFEB)^[7,8]^, respectively. Conversely, nutrient deprivation or metabolic stress rapidly attenuate mTORC1 activity, shifting the cellular metabolic status from growth toward survival. This transition limits macromolecule synthesis while facilitating autophagic degradation, enabling cell adaptation under unfavourable conditions.

### 2.3 The mechanism of mTORC1 activation

**Figure 1:**
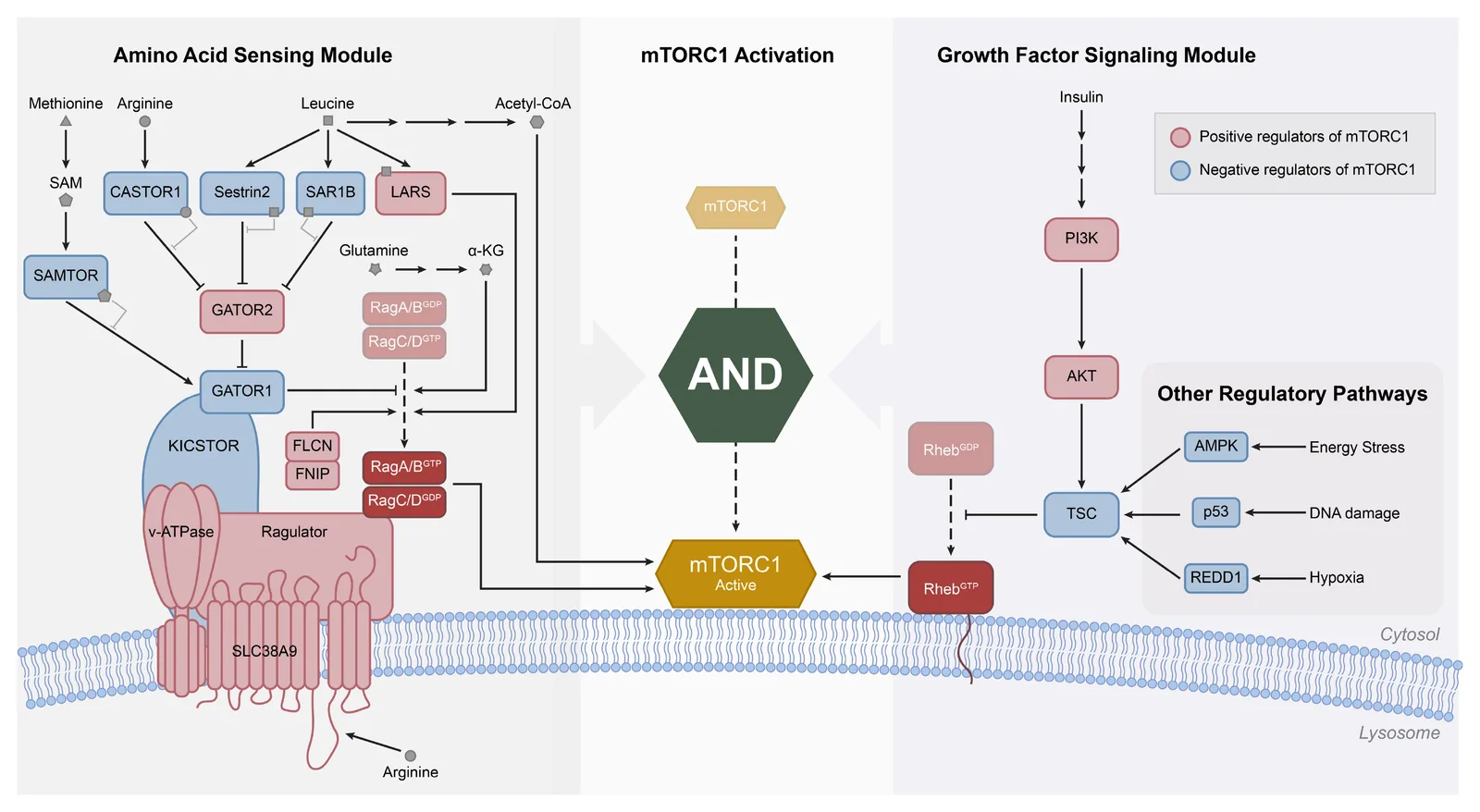
mTORC1 activation by nutrient sensing and growth factor signaling. mTORC1 activation requires the coordinated input of amino acid sufficiency and growth factor signaling, operating as an “AND” gate. To ensure both conditions are met, mTORC1 must first translocate to the lysosomal surface by anchoring onto the Rag GTPases, a step gated by amino acid availability. Cytosolic amino acids are detected by direct sensors, including CASTOR1 (arginine), Sestrin2, SAR1B, and LARS (leucine), that converge on the GATOR1-GATOR2 axis, which modulates the Rag-Ragulator complex. In addition, amino acid availability can be sensed by the FLCN/FNIP complex, which regulates Rag complex configuration since it is a GAP for Rag C/D. Metabolic derivatives of amino acids can also regulate mTORC1 via distinct, Rag-dependent or Rag-independent mechanisms. Lysosomal amino acids are communicated via the lysosomal transmembrane protein SLC38A9 to the Ragulator-Rag machinery. Once recruited to the lysosome, mTORC1 can be activated by GTP-bound Rheb, whose loading state is governed by the TSC complex, the principal brake on mTORC1 that integrates growth factor signaling and stress-responsive inputs including energy status (AMPK), DNA damage (p53), and hypoxia (REDD1). Thus, full kinase activation occurs only when both arms are simultaneously engaged. Positive regulators are indicated in red and negative regulators in blue. mTORC1: mechanistic target of rapamycin complex 1; SAM: S-adenosylmethionine; SAMTOR: SAM sensor upstream of mTORC1; CASTOR1: cellular arginine sensor for mTORC1 subunit 1; SAR1B: secretion-associated Ras-related GTPase 1B; LARS: leucyl-tRNA synthetase 1, cytosolic; GATOR1/2: GAP activity toward Rags complex 1/2; KICSTOR: KPTN–ITFG2–C12orf66–SZT2 complex; FLCN: folliculin; α-KG: α-ketoglutarate; v-ATPase: vacuolar-type H^+^-ATPase; Rag: Ras-related GTP-binding protein; Ragulator: Ragulator complex (or late endosomal/lysosomal adaptor and MAPK and mTOR activator, LAMTOR); SLC38A9: solute carrier family 38 member 9; Rheb: Ras homolog enriched in brain; PI3K: phosphoinositide 3-kinase; AKT: protein kinase B; TSC: tuberous sclerosis complex; AMPK: AMP-activated protein kinase; p53: tumor protein p53; REDD1: regulated in development and DNA damage responses 1.

Given its central role in cellular growth, mTORC1 activity must be tightly controlled. Lysosomes, as degradative organelles and major reservoirs of recycled amino acids, serve as key signaling platforms during mTORC1 activation^[55]^. mTORC1 activation canonically requires its translocation to the lysosomal membrane, where its master activator - the small GTPase Rheb (Ras homologue enriched in brain), and its recruitment machinery, the Rag GTPases converge (Figure 1)^[6,26,34]^.

Rheb functions as an essential mTORC1 activator controlled by several growth factor signaling pathways, such as the insulin/ insulin-like growth factor-1 (IGF-1) pathway^[38,56]^. These mitogen-dependent pathways inhibit the tuberous sclerosis complex (TSC)^[39,57]^, a GTPase activating protein (GAP) for Rheb^[58–61]^. Inhibition of TSC leads to the accumulation of GTP-bound Rheb that directly binds and stimulates mTORC1 kinase activity^[62,63]^. Rag GTPases relay a second class of environmental cues, nutrient availability, through their nucleotide loading state^[6,38]^. Rag GTPases form obligate heterodimers composed of RagA or RagB (RagA/B) paired with RagC or RagD (RagC/D)^[64,65]^. mTORC1 lysosomal recruitment occurs only when RagA/B is GTP-bound and RagC/D is GDP-bound^[35,36]^. Through this two-tiered mechanism, mTORC1 coordinates growth factor signaling and nutrient sufficiency, ensuring that its activity is engaged only when both requisites are met.

### 2.4 Amino acid sensing by mTORC1

Beyond their role as the building blocks of proteins, amino acids supply critical energy and carbon sources through their metabolism, making their availability a major determinant of mTORC1 activity. Multiple amino acids, including leucine^[66]^, arginine^[67,68]^, methionine^[69,70]^, threonine^[71]^, glutamine^[72,73]^, asparagine^[73]^, histidine^[74]^, and valine^[75]^, have been shown to regulate mTORC1 signaling through diverse yet convergent mechanisms over the last decade, with each amino acid often leveraging multiple routes to precisely tune mTORC1 activity.

Under basal conditions, extracellular amino acids are taken up and metabolised by cells. These amino acids or their derivatives are sensed by specialised sensors that transmit signals to the Rag GTPases via GATOR1 andGATOR2 ^[6,38,76^. GATOR1, composed of DEP domain containing 5 (DEPDC5), Nitrogen permease regulator 2-like protein (NPRL2), and NPRL3, inactivates mTORC1 by exerting GAP activity toward RagA/B^[77–79]^. In contrast, GATOR2, composed of Meiosis regulator for oocyte development (MIOS), WD repeat-containing protein 24 (WDR24), WDR59, SEH1-like protein (SEH1L), and SEC13 homolog, nuclear pore and COPII coat complex component (SEC13), counteracts GATOR1 function to permit mTORC1 lysosomal localisation^[77,79]^. Notably, GATOR2 serves as a major convergence point, directly regulated by the leucine sensors Sestrin2^[80,81]^ and secretion associated Ras related GTPase 1B (SAR1B)^[82]^, as well as the cytosolic arginine sensor for mTORC1 subunit 1 (CASTOR1)^[67,68]^.

In addition to cytosolic amino acids, lysosomal amino acid levels, especially lysosomal arginine levels, also contribute to mTORC1 activity via the Ragulator complex, a second regulator for Rag GTPases. Ragulator is a pentameric assembly comprising LAMTOR1-5 (also known as p18, p14, MP1, C7ORF59, and HBXIP)^[83–85]^. It is anchored to the lysosomal membrane and functions as a guanine-nucleotide exchange factor (GEF) toward RagA/B^[84]^. Lysosomal amino acids regulate Ragulator’s GEF activity through two lysosome-localised proteins: the vacuolar H^+^-ATPase (v-ATPase)^[86]^, and the solute carrier family 38 member 9 (SLC38A9)^[87–89]^, both of which physically bind to the Ragulator-Rag complex. This multiprotein assembly then promotes Ragulator-mediated GTP loading onto RagA/B, thereby maintaining the active Rag configuration required for mTORC1 lysosomal recruitment and subsequent activation by Rheb^[84,85]^.

Several additional mechanisms of amino acid-regulated mTORC1 signaling have also been described. Notably, the folliculin (FLCN)-FNIP complex has been identified as a GAP for RagC/D at the lysosomal surface, facilitating mTORC1 activation when amino acids are presented, which represents an important non-canonical regulatory pathway^[55,90,91]^.

### 2.5 Canonical leucine sensing by leucine sensors

Although many amino acids are capable of regulating mTORC1, certain species exert more pronounced effects, such as glutamine, leucine, and arginine^[76,92]^. Several early studies have established leucine as the most potent amino acid regulator of mTORC1^[66,93,94]^, which is sensed by multiple parallel sensors, including Sestrin2, SAR1B, and leucyl-tRNA synthetase (LARS)^[2,92]^. Leucine directly binds to Sestrin2 and SAR1B and disrupts their interaction with different subunits of GATOR2 (SEH1L and MIOS), relieving GATOR2 inhibition thereby permitting mTORC1 lysosomal translocation for its activation^[80–82]^. In parallel, LARS senses leucine and catalyses leucyl aminoacylation, which enhances its non-canonical GAP activity toward RagD to promote the GDP-bound state required for mTORC1 activation^[95–98]^. In contrast, leucine deprivation inhibits mTORC1 activity via Sestrin2- and SAR1B-mediated GATOR2 inhibition, as well as uncharged LARS-mediated Rag GTPase inhibition. Virtually of all the studies on the above sensors have been done mainly in HEK293 cells.

### 2.6 Leucine-derived AcCoA regulates mTORC1 activity

**Figure 2:**
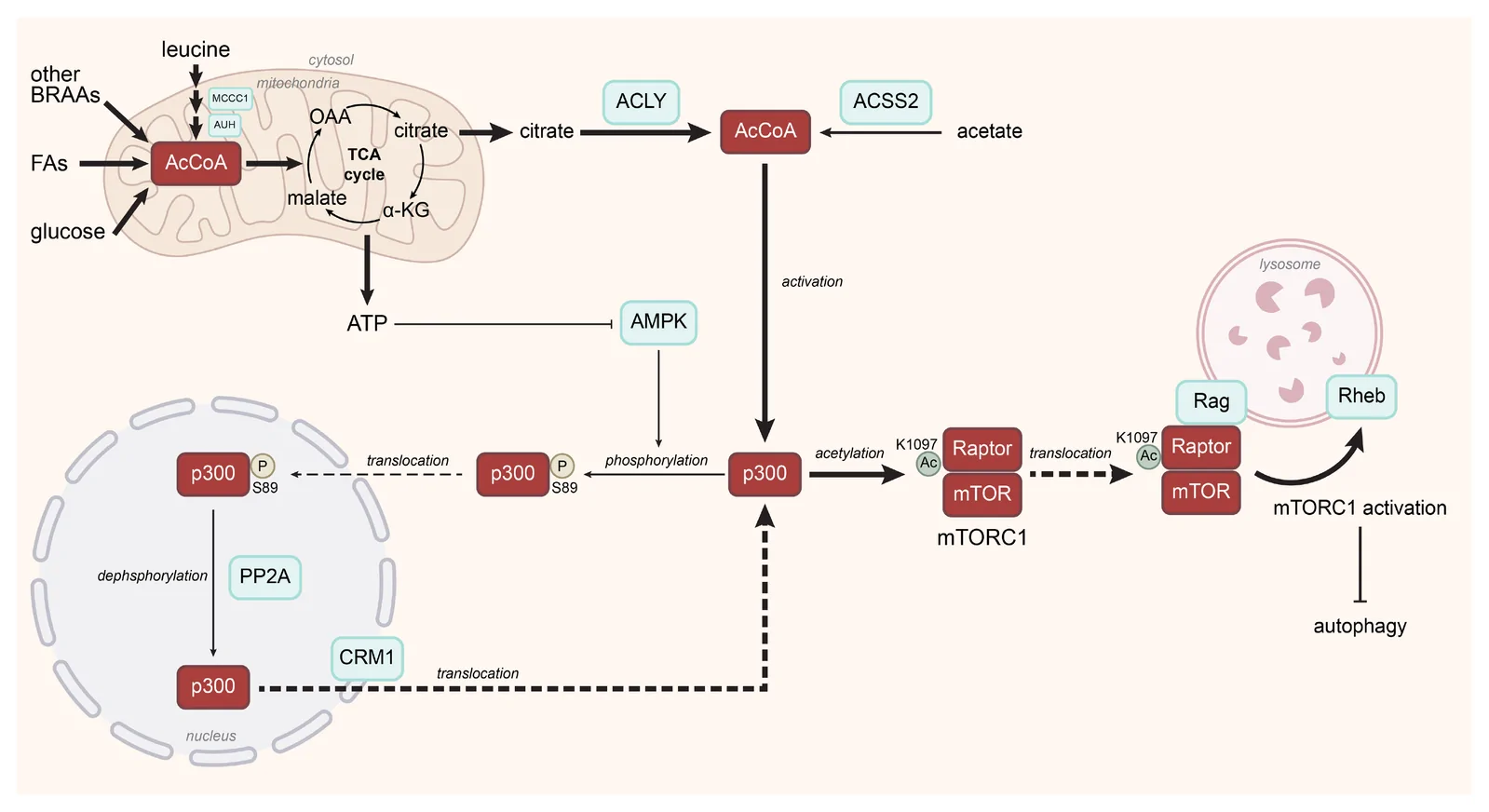
Acetyl-CoA activates mTORC1 via p300-mediated Raptor acetylation. Nutrients, including leucine, are broken down into AcCoA and other intermediates to sustain mitochondrial TCA cycle flux, generating ATP and citrate as metabolic outputs. Citrate is exported to the cytoplasm and converted to AcCoA by ACLY. In parallel, intracellular acetate serves as an additional AcCoA source mediated by ACSS2. Cytosolic AcCoA acts as the substrate for p300, a lysine acetyltransferase that shuttles between the nucleus and cytoplasm. Under nutrient-replete conditions, high ATP production reduces the AMP:ATP ratio, thereby attenuating AMPK activity. This reduced AMPK signaling diminishes phosphorylation of p300 at Serine 89 (S89), a modification that otherwise promotes its nuclear import. Nuclear p300 is dephosphorylated by PP2A, enabling its export back to the cytoplasm by CRM1. Cytosolic p300 catalyses acetylation of Raptor at Lysine 1097 (K1097) using AcCoA as the acetyl donor, which enhances Raptor interaction with Rag GTPases, promotes mTORC1 lysosomal translocation, and thereby allows Rheb-mediated mTORC1 activation and subsequent suppression of autophagy. Thick arrows and red components indicate pathways active under nutrient-replete conditions. Thin arrows denote inactive processes. Dashed lines indicate translocation, and lines with flat heads indicate inhibition. BRAAs: branched-chain amino acids; FAs: fatty acids; AcCoA: acetyl-Coenzyme A; MCCC1: 3-methylcrotonyl-CoA carboxylase 1; AUH: AU RNA-binding methylglutaconyl-CoA hydratase; TCA cycle: tricarboxylic acid cycle; OAA: oxaloacetate; α-KG, α-ketoglutarate; ACLY: ATP-citrate lyase; ACSS2: acetyl-CoA synthetase 2; p300: E1A-binding protein p300; AMPK: adenosine monophosphate-activated protein kinase; PP2A: protein phosphatase 2A; CRM1: chromosome region maintenance 1 (exportin-1); Raptor: regulatory-associated protein of mTOR; Rag: Ras-related GTP-binding protein; Rheb: Ras homolog enriched in brain; mTORC1: mechanistic target of rapamycin complex 1.

Beyond direct sensing by canonical leucine sensors, leucine also contributes to mTORC1 activity through AcCoA, a downstream intermediate in leucine metabolism^[2,25]^. Leucine-derived AcCoA intersects with protein acetylation to regulate mTORC1 activity, particularly in highly metabolically active neurons^[18,25]^. This mechanism broadens the scope of amino acid-mTORC1 signaling from direct nutrient sensing to metabolic regulation (Figure 2). Subsequent studies demonstrated that disruption of this AcCoA-mTORC1 axis contributes to neurodegenerative pathology and highlighted its potential as a therapeutic target.

AcCoA is a central carbon metabolite that fuels the tricarboxylic acid (TCA) cycle. It also supports lipid biosynthesis and serves as an acetyl donor for protein acetylation^[99]^. Under basal conditions, nutrient sufficiency sustains mitochondrial TCA cycle activity, which maintains the cytosolic AcCoA pool through two key enzymes: ATP-citrate lyase (ACLY) and acetyl-CoA synthetase 2 (ACSS2)^[99]^. In parallel, leucine catabolism also generates AcCoA as a final product, providing an additional critical source of cytosolic AcCoA. This accumulation of AcCoA enhances the activity of the acetyltransferase p300 by providing acetyl groups and facilitating its translocation from the nucleus to the cytosol. Activated cytosolic p300 directly acetylates Raptor at K1097, strengthening the interaction of Raptor with the Rag GTPases for subsequent lysosomal translocation and activation of mTORC1. Consequently, leucine-driven mTORC1 signaling suppresses autophagy^[18,25]^.

Conversely, amino acid starvation, particularly leucine limitation, reduces cytosolic AcCoA levels, diminishes cytoplasmic p300 activity, and favors its nuclear redistribution. This reduces Raptor acetylation, weakens the Raptor-Rag interaction, attenuates mTORC1 signaling, and permits autophagy induction. Collectively, these findings incorporate leucine metabolism, AcCoA-dependent acetylation, and mTORC1 regulation, revealing a metabolite-driven regulatory layer on amino acid sensing and mTORC1 signaling^[18,25]^. This process operates in many cell types including primary neurons and glia, where it appears to be dominant compared to the more traditional leucine sensors. However, the traditional leucine sensors are dominant in HEK293 cells and mouse embryonic fibroblasts, where leucine depletion results in very little effect on AcCoA levels, compared to cells which “sense” leucine via AcCoA^[25]^. This leucine-AcCoA-p300-mTORC1-autophagy pathway that we described is consistent with previous observations relating AcCoA to autophagy^[100]^ and subsequent studies that support the relevance of this pathway in zebrafish^[101]^ and mice^[102]^.

We found that this mechanism likely operates in cells after about 60 minutes of amino acid starvation^[103]^, after which AcCoA levels have likely declined sufficiently to impact p300 specific activity. In the first hour after amino acid starvation, p300 cytoplasmic activity is reduced because it is shuttled into the nucleus, which is mediated by phosphorylation by the energy sensor AMPK. When nutrients are added back to such starved cells, then p300 is dephosphorylated and shuttled back into the cytoplasm from the nucleus by an exportin-dependent mechanism^[103]^. Interestingly, this p300 shuttling mechanism regulating mTORC1 is not restricted to leucine starvation but also responds to glucose deprivation. It also acts in all 9 cell lines tested, including mouse embryonic fibroblasts and HEK293 cells^[103]^. Importantly, recent studies from our group demonstrated that aberrant mTORC1 hyperactivation, a common feature in neurodegenerative diseases, is at least partially caused by perturbations in the AcCoA-p300-mTORC1 axis^[24,103]^. In Hutchinson–Gilford progeria syndrome (HGPS), defective nucleocytoplasmic shuttling of p300 increases its cytoplasmic abundance, thereby enhancing Raptor acetylation, driving mTORC1 hyperactivation^[103]^. In Parkinson’s disease models with the A53T alpha-synuclein mutation or alpha-synuclein triplication, there is abnormal activation of ACLY, which leads to cytosolic AcCoA accumulation and mTORC1 hyperactivation, which ultimately contributes to neurodegenerative progression^[24]^. Notably, pharmacological inhibition of ACLY attenuates pathological mTORC1 activation and ameliorates disease associated phenotypes in cell, organoid, zebrafish and mouse models, underscoring the importance of this AcCoA-p300-mTORC1 signaling pathway in disease pathology and highlighting its therapeutic potential^[24]^.

### 2.7 mTORC1 regulation by other amino acid-derived metabolites

Apart from leucine, other amino acids can also promote mTORC1 activity through their metabolic derivatives. For instance, methionine acts via its downstream product S-adenosyl-L-methionine (SAM), which binds to the SAM sensor upstream of mTORC1 (SAMTOR), an inhibitory interactor of GATOR1, releasing it from GATOR1. High SAM disrupts binding of SAMTOR to the GATOR/KICSTOR (KPTN, ITFG2, C12orf66, and SZT2-containing regulator) complex, thereby facilitating mTORC1 activation^[69,70]^. Similarly, glutamine metabolism generates α-ketoglutarate through glutaminolysis, and this metabolite activates mTORC1 in a Rag-dependent manner in the presence of leucine^[72]^.

## 3 Dysregulation of Metabolic Signaling in Neurodegeneration

Neurons are post-mitotic, metabolically demanding cells that depend on sustained proteostatic surveillance across decades of life^[17,104]^. Under normal physiological conditions, mTORC1 activity in neurons is tightly regulated in a spatially compartmentalised manner: it promotes ribosomal biogenesis and local protein synthesis at synaptic terminals to support long-term potentiation and dendritic remodelling^[15]^, coordinates axonal growth and branching during development^[105]^, and gates autophagic flux to maintain organelle quality control, including the selective removal of depolarised mitochondria through mitophagy^[106–108]^. Critically, mTORC1 activity in healthy neurons undergoes dynamic oscillation, activating in response to nutrient and growth factor availability and downregulating during metabolic stress to permit autophagic clearance, a balance that is essential given the neuron’s inability to dilute damaged proteins through cell division^[4,56]^.

The failure of this dynamic regulation is a defining feature of the transition from healthy brain aging to neurodegeneration^[4]^. While mTORC1 is essential for neural elongation and branching, synaptic plasticity, and neural differentiation^[19,104,108]^, its chronic hyperactivation or its failure to downregulate during metabolic stress drives pathology by actively suppressing the autophagic removal of toxic aggregate-prone proteins^[109]^ and other autophagic substrates like dysfunctional organelles. (Table 1).

### 3.1 Alzheimer’s disease

Alzheimer’s disease is the most common neurodegenerative disorder, which is defined by extracellular amyloid-β (Aβ) plaques, intracellular hyperphosphorylated tau tangles, synaptic dysfunction, and progressive cognitive decline^[110,111]^. Multiple studies report basal hyperactivation of the PI3K–Akt–mTORC1 axis in AD brain and transgenic models, with increased phosphorylation of Akt, mTOR, p70S6K, and 4E-BP1^[112–115]^. This persistent signaling correlates with reduced autophagic markers and impaired flux, limiting clearance of Aβ and tau^[109,116]^. Mechanistically, Aβ can promote mTORC1 activation via PTEN modulation, PRAS40 phosphorylation, and insulin resistance mediated by IRS-1 inhibitory phosphorylation^[4,117,118]^. AMPK signaling is also altered in a stage-dependent manner, further disrupting metabolic flexibility^[114,119]^. Functionally, mTORC1 hyperactivation both suppresses autophagy and perturbs synaptic protein translation^[116,120]^. Genetic or pharmacological mTOR inhibition enhances autophagic flux, reduces amyloid and tau burden, and improves cognition in several models^[109,121–124]^. Thus, AD illustrates how maladaptive persistence of mTORC1 activity limits proteostatic adaptation under chronic metabolic stress.

### 3.2 Parkinson’s disease

Parkinson’s disease is characterized by degeneration of dopaminergic neurons and accumulation of α-synuclein in Lewy bodies^[125]^. Mitochondrial dysfunction and oxidative stress are central pathogenic drivers, particularly in metabolically demanding nigral neurons^[4,126]^. mTOR signaling in PD is highly context-dependent^[4]^. Acute oxidative stress models often show Akt–mTOR suppression, partly via REDD1 induction and ROS-mediated kinase damage^[127]^. In contrast, α-synuclein–driven and chronic metabolic models frequently exhibit mTORC1 hyperactivation with impaired autophagic flux, suggesting a failure to recalibrate nutrient sensing^[109,128]^. Recent work^[24]^ provides mechanistic resolution to this discrepancy by identifying AcCoA as a metabolic rheostat controlling mTORC1 via p300-mediated acetylation of Raptor. In dopaminergic neurons, altered AcCoA dynamics sustain p300 activity, stabilize mTORC1 signaling, and prevent appropriate stress-induced suppression. This acetylation-dependent persistence impairs autophagic clearance of α-synuclein and heightens proteotoxic vulnerability. Modulating the AcCoA–p300 axis restores physiological mTORC1 oscillation and improves neuronal resilience^[24]^. Thus, PD highlights a critical principle where neuronal fate depends on correct metabolic interpretation, and AcCoA–driven mTORC1 persistence links mitochondrial dysfunction directly to impaired autophagy.

### 3.3 Huntington’s disease

Huntington’s disease is caused by CAG repeat expansion mutations in HTT, producing polyglutamine-expanded mutant huntingtin (mHtt)^[129]^. Early synaptic dysfunction and progressive striatal neurodegeneration define disease progression^[130]^. In many HD models, mTORC1 hyperactivation contributes to pathology by suppressing autophagy and promoting excessive protein synthesis, thereby exacerbating proteotoxic stress^[129,131]^. Pharmacological or genetic mTOR inhibition enhances autophagic clearance of mHtt aggregates and improves phenotypes in several experimental systems^[132,133]^. Activation of mTORC1 via Tuberous Sclerosis Complex-1 (TSC1) deletion or Rheb signaling accelerates disease features, supporting a pathogenic role for sustained mTORC1 activity^[134,135]^. However, stress-induced REDD1 upregulation and compensatory Akt/mTORC2 activation have also been observed^[136]^, indicating dynamic remodeling rather than uniform pathway activation. HD therefore reflects an imbalance between anabolic drive and clearance capacity, where inappropriate mTORC1 persistence amplifies aggregation burden.

### 3.4 ALS and frontotemporal dementia (FTD)

ALS and frontotemporal dementia (FTD) frequently converge on TDP-43 proteinopathy and lysosomal dysfunction^[137–139]^, but mTOR regulation varies across genetic contexts^[140]^. In C9ORF72-linked ALS/FTD, loss of C9ORF72 disrupts lysosomal nutrient sensing and alters mTORC1 signaling, linking autophagy control to lysosome-associated metabolic regulation^[141–143]^. In SOD1-based ALS models, motor neuron survival can depend on maintaining mTOR activity, and rapamycin treatment has worsened outcomes in some systems^[144,145]^. This may be because autophagy inhibition reduces glial inflammation in such models^[146]^. In contrast, tau-driven FTD models showing mTOR overactivation respond to mTOR inhibition with reduced pathology and improved behavioural performance^[147]^. Importantly, mTOR-independent autophagy activation (e.g., trehalose) has demonstrated neuroprotective effects in certain ALS/FTD contexts^[148]^, suggesting that autophagy enhancement and mTOR inhibition are not equivalent strategies. ALS/FTD underscores that optimal metabolic intervention depends on whether disease is driven primarily by bioenergetic insufficiency, lysosomal dysfunction, or aggregation stress.

### 3.5 Non-cell-autonomous regulation of neuronal mTORC1

In the prodromal stages of most neurodegenerative diseases, microglia are activated^[149,150]^. These cells secrete chemokines which inhibit neuronal autophagy^[151]^. These effects can be accounted for by microglial chemokine (C-C motif) ligand 3 (CCL3), CCL4 and CCL5, which agonise neuronal C-C chemokine receptor type 5 (CCR5), a G protein-coupled receptor (GPCR), which stimulates mTORC1 and reduces autophagosome activity^[151]^. This process was also observed in mouse models of Huntington’s disease and tauopathy, where CCR5 knockout or inhibition with the FDA-approved drug maraviroc rescued the mTORC1 hyperactivation and impaired autophagy and ameliorated signs of disease^[151,152]^. This leads to the possibility that maraviroc could be repurposed for the treatment of these conditions^[151]^.

## 4 Conclusion

mTORC1 regulation in neurons is increasingly recognised as a dynamic and finely tuned system rather than a simple growth switch. In post-mitotic neurons, mTORC1 functions as a metabolic rheostat that coordinates the energetic demands of synaptic plasticity with the need for efficient autophagic quality control.

Recent advances highlight that nutrient sensing extends beyond canonical amino acid pathways to include metabolic flux integration. The AcCoA–p300–Raptor axis links mitochondrial metabolism to mTORC1 activity through acetylation, providing a mechanism by which neurons adjust anabolic signaling to cellular energy state. Across neurodegenerative diseases, including AD, PD, and HD, persistent mTORC1 hyperactivation emerges as a common pathogenic feature, promoting protein synthesis while impairing autophagic clearance of toxic aggregates. In addition, non-cell autonomous signals, such as microglial chemokines acting through neuronal CCR5, further contribute to dysregulated mTORC1 signaling within the diseased brain. It is interesting to note that epilepsy is much more frequent in Alzheimer’s disease and Parkinson’s disease^[153]^. Since mTORC1 hyperactivation can cause epilepsy^[154]^, it is tempting to speculate that this mechanism may contribute to epilepsy in some patients with neurodegeneration. While we have focused on nutrient and metabolic signals that increase mTORC1 activity in neurodegeneration, it is also worth considering that mitochondrial defects are often associated with neurodegeneration. In such cases, it is relevant to consider data suggesting that mitochondrial inhibition typically leads to AMPK activation, which decreases mTORC1 activity. Thus, it is possible that mTORC1 activity may switch from hyperactivity to hypoactivity (or vice versa) as disease progresses in individual neurons/glia.

Looking ahead, therapeutic strategies targeting upstream metabolic regulators, including modulating AcCoA production, may offer additional approaches to rebalance neuronal proteostasis. A deeper understanding of neuron-specific nutrient-sensing mechanisms will be critical for developing effective disease-modifying interventions.

## Figures and Tables

**Table 1:** mTORC1 dysregulation in neurodegenerative diseases.

Disease	mTORC1 directionality	Key upstream drivers	Autophagy status	Translational implication	References
**AD**	Predominantly hyperactivated (stagedependent)	Aβ, insulin resistance (IRS-1), PTEN modulation, AMPK imbalance	Reduced autophagic flux	mTORC1 inhibition or AMPK activation may enhance Aβ/tau clearance	[109,110,112–114]
**PD**	Context-dependent; chronic metabolic models show persistence	Mitochondrial dysfunction, AcCoA-p300 acetylation, α-synuclein	Impaired stress- induced induction	Target upstream metabolic checkpoints (AcCoA-p300 axis) rather than global inhibition	[24,109]
**HD**	Often hyperactivated; dynamic remodeling	mHtt aggregation, translational stress, REDD1 induction	Suppressed clearance in hyperactive states	Autophagy induction beneficial in many models; stage-specific targeting required	[132,134,136]
**ALS/FTD**	Highly mutation-specific	C9ORF72 lysosomal dysfunction, SOD1 stress, tau/TDP-43 pathology	Variable; sometimes increased but ineffective	mTOR-independent autophagy activation may be preferable in some contexts	[141,144,146,148,151]
**Neuroinflammation**	Non-cell autonomous activation	Microglial CCL3/4/5 acting on neuronal CCR5	Inflammation- driven suppression of autophagy	Maraviroc (CCR5 inhibitor)	[152]

AD: Alzheimer’s disease; Aβ: amyloid beta; PTEN: phosphatase and tensin homolog; AMPK: AMP-activated protein kinase; PD: Parkinson’s disease; AcCoA: acetyl-Coenzyme A; p300: E1A-associated protein p300 (EP300); HD: Huntington’s disease; mHtt: mutant huntingtin; REDD1: regulated in development and DNA damage response 1; mTORC1: mechanistic target of rapamycin complex 1; ALS/FTD: amyotrophic lateral sclerosis/frontotemporal dementia; C9ORF72: chromosome 9 open reading frame 72; SOD: superoxide dismutase; TDP-43: TAR DNA-binding protein 43; CCL3/4/5: C-C motif chemokine ligand 3/4/5; CCR5: C-C chemokine receptor 5.

## Data Availability

Not applicable.
